# Serum iron: a new predictor of adverse outcomes independently from serum hemoglobin levels in patients with acute decompensated heart failure

**DOI:** 10.1038/s41598-021-82063-0

**Published:** 2021-01-27

**Authors:** Tomoya Ueda, Rika Kawakami, Kazutaka Nogi, Maki Nogi, Satomi Ishihara, Yasuki Nakada, Tomoya Nakano, Yukihiro Hashimoto, Hitoshi Nakagawa, Taku Nishida, Kenji Onoue, Tsunenari Soeda, Satoshi Okayama, Makoto Watanabe, Yoshihiko Saito

**Affiliations:** Cardiovascular Medicine, Nara Medical University, 840 Shijo, Kashihara, Nara, 634-8522 Japan

**Keywords:** Biomarkers, Cardiology, Risk factors

## Abstract

Iron is an essential trace element in the body. However, in heart failure (HF), iron is only recognized as the cause of anemia. Actually, iron itself affects myocardial exercise tolerance and cardiac function via mitochondrial function. Therefore, it is necessary to clarify the pathological significance of iron in acute HF, irrespective of concomitant anemia. We investigated the impact of serum iron level at discharge on the prognosis of 615 patients emergently admitted with acute decompensated HF (ADHF). Patients were divided into two groups according to the median level of serum iron (62 µg/dL). The endpoint was the composite outcome, which included all-cause mortality and readmission for HF. During the mean follow-up period of 32.1 months, there were 333 events. Kaplan–Meier analysis showed that the incidence of the composite outcome was significantly higher in the Low iron group (P < 0.0001). In the multivariate analysis adjusted with factors including hemoglobin and ferritin levels, low serum iron was an independent predictor for the composite outcome (hazard ratio, 1.500; 95% confidence interval, 1.128–1.976; P = 0.0044). Low serum iron was an independent predictor of poor prognosis in ADHF, irrespective of hemoglobin or ferritin level, providing a new concept that iron may play a role in the pathophysiology of ADHF via non-hematopoietic roles.

## Introduction

Heart failure (HF) is a major public health issue worldwide. Recent guidelines on acute and chronic HF have classified HF into three groups according to left ventricular ejection fraction (LVEF): HF with reduced EF (HFrEF; LVEF < 40%), mid-range EF (HFmrEF; 40% ≤ LVEF < 50%), and preserved EF (HFpEF; 50% ≤ LVEF)^1, 2^. Despite optimal conventional therapy, patients with HF still show less improvement in symptoms as well as high readmission and mortality rates^2–4^. One reason for this may be the occurrence of comorbidities. In particular, it is well known that anemia is a strong factor associated with prognosis in HF, and iron is one of the major causes of anemia. Interestingly, iron is not only a factor of anemia, but also plays a crucial role in cardiac and skeletal muscle metabolism via mitochondrial function and affects myocardial exercise tolerance and cardiac function. However, little is known about the relationship between HF and iron itself beyond the cause of anemia. Therefore, it is necessary to clarify the pathological significance of iron in acute HF, irrespective of concomitant anemia.

Iron is an essential trace element in the body and is crucial for oxygen transport, delivery, and utilization. The regulation of systemic iron balance is also important for the homeostasis of myocytes and skeletal muscle cells, which depend on iron for their function and structural integrity^5, 6^. Interestingly, both iron deficiency (ID) and iron overload adversely affect cardiovascular health. ID is present in approximately 50% of patients with HF, regardless of whether it is HFrEF or HFpEF; ID is one of the representative comorbidities of HF^7–9^.

Previous studies have investigated the association between ferritin and chronic HF and demonstrated a significant association between ID, diagnosed based on ferritin levels, and poor outcomes^7, 12–14^. Ferritin is most frequently used as an indicator of ID, but some patients are diagnosed with functional ID despite high ferritin levels. Regarding this fact, the action of hepcidin has a great influence. Because hepcidin plays an important role in the regulation of iron metabolism, including the absorption of iron and liberation of iron stores within the body. Interestingly, the activity of hepcidin is enhanced by inflammation and previous studies have demonstrated that acute HF is associated with systemic inflammation^10, 11^. In short, the increasing hepcidin activity by inflammation in HF may lead to the development of functional ID despite high ferritin levels. Therefore, to better understand the pathophysiology of iron metabolism in HF, not only ferritin but also iron itself should be studied in more detail and the association between iron levels and clinical outcomes should be explore. In this study, we examined the impact of serum iron levels in patients with acute decompensated HF (ADHF).

## Results

### Baseline characteristics

As shown in Table 1, the mean age of the 615 patients was 74.3 ± 12.0 (mean ± SD) years, and the proportion of men was 56.9%. Median level of serum iron and ferritin were 62.0 (44.0–88.0) µg/dL and 107 (51–203) µg/L (medians (25th and 75th percentile)), respectively. To investigate the impact of serum iron level on the prognosis of ADHF, we divided patients into two groups according to the median serum iron level at discharge (62 mg/dL). Table 1 shows baseline clinical characteristics of patients in the Low and High iron groups. Compared to patients in the High iron group, those in the Low iron group were significantly older. There were no significant differences in the cause of HF or proportion of comorbidities between the groups, except for diabetes mellitus. The rate of ID based on ferritin level, which was defined as ferritin level < 100 ug/L or 100–299 ug/L if TSAT is < 20%, was significant higher in the Low iron group than in the High iron group. Moreover, vital signs at discharge and LVEF were similar, but the left ventricular end-diastolic diameter was shorter in the Low iron group. Regarding laboratory findings, the hemoglobin level and eGFR were lower and C-reactive protein (CRP) and BNP levels were higher in the Low iron group. TIBC, TSAT, ferritin and transferrin levels were lower in the Low iron group. The proportion of patients treated with angiotensin-converting enzyme inhibitors/angiotensin receptor blockers, β-blockers, or diuretics was similar in both groups at discharge, except for mineralocorticoid receptor blockers. With regard to the rate of the rate of administration of iron tablets, it was significantly higher in the Low iron group than in the High iron group.

**Table 1:** Baseline characteristics of HF patients in the low vs. high iron group.

Characteristic	Total (n = 615)	Low iron (n = 307)	High iron (n = 308)	P value
**Demographic**				
Age, years	74.3 ± 12.0	76.3 ± 10.8	72.2 ± 12.8	< 0.0001
Male, %	56.9	53.4	60.4	0.0808
BMI, kg/m^2^	23.3 ± 4.4	23.0 ± 4.1	23.7 ± 4.6	0.1110
**Cause of HF, %**				
Ischemic	33.5	34.9	32.1	0.4764
Valvular	17.4	18.9	15.9	0.3289
Dilated cardiomyopathy	15.5	13.0	17.9	0.0970
Hypertensive	7.0	6.5	7.5	0.6430
**Medical history, %**				
Hypertension	74.3	76.9	71.8	0.1460
Diabetes mellitus	41.3	47.2	35.4	0.0028
Dyslipidemia	43.1	45.9	40.3	0.1557
Smoking	19.7	16.9	22.4	0.0879
ID based on ferritin level	57.4	40.5	74.6	< 0.0001
**NYHA class on admission, %**				
III or IV	91.5	91.2	91.9	0.7625
**Vital sign at discharge**				
Systolic blood pressure, mmHg	111.0 ± 17.8	112.0 ± 18.6	110.1 ± 16.9	0.3052
Heart rate, beats/minute	71.4 ± 11.9	71.7 ± 11.7	71.1 ± 12.0	0.5946
**Echocardiographic parameters**				
LVEF, %	46.7 ± 16.0	47.6 ± 16.2	45.9 ± 15.7	0.1872
HFpEF, %	40.7	42.7	38.6	0.2986
LVEDD, mm	52.4 ± 9.3	51.3 ± 9.1	53.4 ± 9.4	0.0017
**Laboratory data at discharge**				
C-reactive protein, mg/dL*	0 .28 (0.10–0.90)	0.50 (0.10–1.30)	0.20 (0.10–0.50)	< 0.0001
Hemoglobin, g/dL	11.5 ± 2.1	10.8 ± 1.7	12.3 ± 2.2	< 0.0001
MCV, fl	93.8 ± 6.7	92.6 ± 6.9	95.0 ± 6.2	< 0.0001
eGFR, mL/min/1.73m^2^	42.5 ± 22.2	37.2 ± 21.0	47.8 ± 22.1	< 0.0001
Sodium, mmol/L	137.9 ± 4.0	138.0 ± 4.0	137.9 ± 4.0	0.7095
Plasma BNP, pg/mL*	272 (145–508)	326 (182–626)	225 (124–425)	< 0.0001
Serum iron, µg/dL*	62.0 (44.0–88.0)	44.0 (33.0–53.0)	88.0 (71.0–110.0)	< 0.0001
TIBC, µg/dL	287.9 ± 66.2	275.5 ± 71.8	300.3 ± 57.6	< 0.0001
TSAT, %	24.8 ± 14.7	16.7 ± 12.5	32.8 ± 12.1	< 0.0001
Ferritin, µg/L	107 (51–203)	86 (40–175)	128 (63–224)	< 0.0001
Transferrin, mg/dL	224.8 ± 52.2	217.5 ± 54.7	231.4 ± 49.0	0.0005
**Medication at discharge, %**				
ACE inhibitors or ARBs	87.3	85.3	89.3	0.1410
MR blockers	43.6	39.1	48.1	0.0249
β-blockers	74.5	71.7	77.3	0.1103
Diuretics	78.5	79.5	77.6	0.5698
Iron tablets	15.0	9.7	20.2	0.0003

ID based on ferritin level is defined as ferritin level < 100 ug/L or 100–299 ug/L if TSAT is < 20%.

*HF* heart failure; *BMI* body mass index; *ID* iron deficiency; *NYHA* New York Heart Association; *LV* left ventricular; *EF* ejection fraction; *HFpEF* heart failure with preserved ejection fraction; *EDD* end-diastolic diameter; *MCV* mean corpuscular volume; *eGFR* estimated glomerular filtration rate; *BNP* B-type natriuretic peptide; *TIBC* total iron-binding capacity; *TSAT* transferrin saturation; *ACE* angiotensin-converting enzyme; *ARB* angiotensin receptor blocker; *MR* mineralocorticoid receptor.

*Data are shown as percentages, means ± standard deviation, or medians (25th and 75th percentile).

### Prognosis and outcome

**Figure 1:**
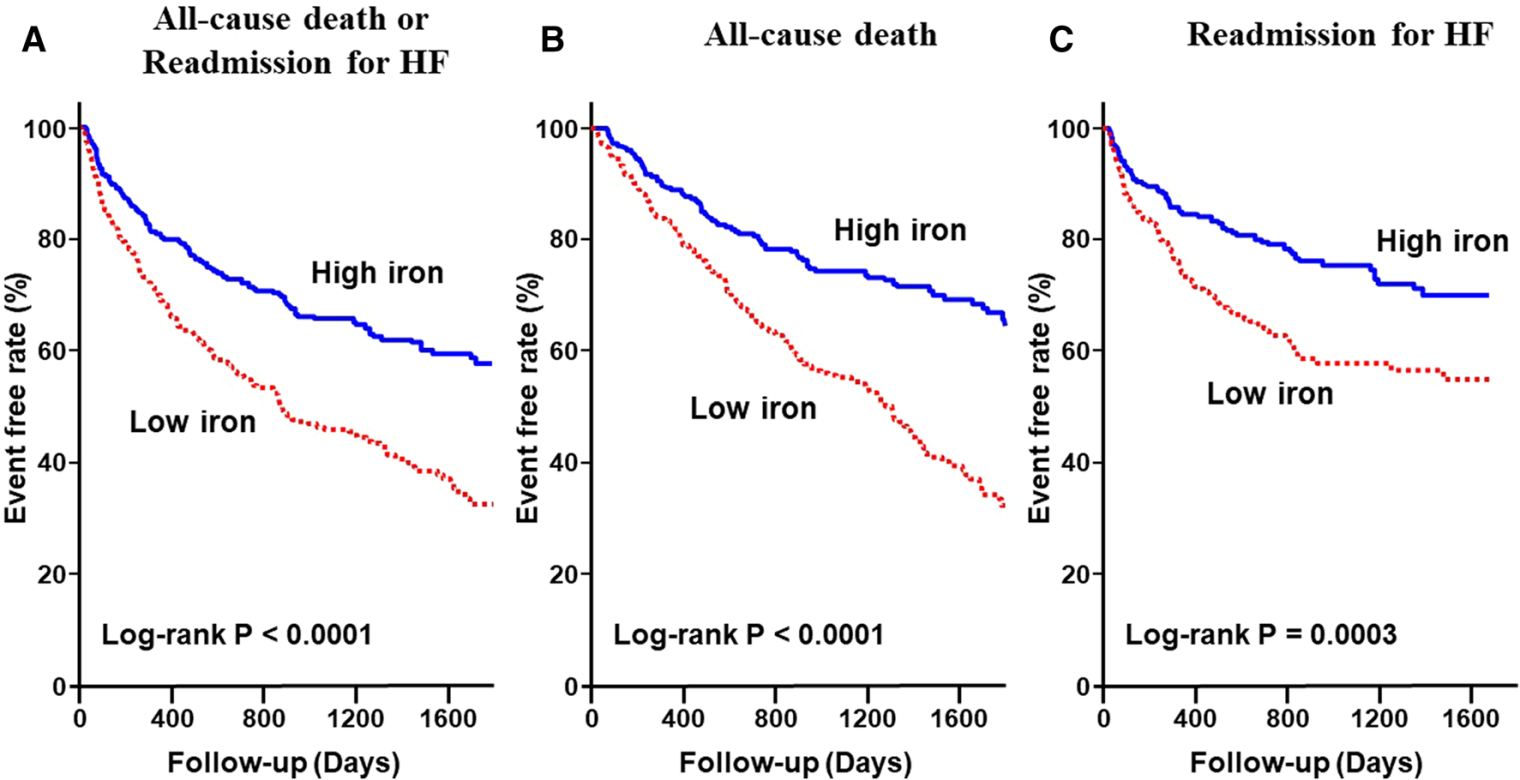
Kaplan–Meier event-free survival curves for (**A**) all-cause mortality or readmission for HF, (**B**) all-cause mortality, and (**C**) readmission for HF in the Low iron group (red line) compared with the High iron group (blue line). HF, heart failure.

During the mean follow-up period of 32.1 months, there were 333 composite outcomes; among these, 153 were all-cause mortality and 180 were readmission for HF. As shown in the Kaplan–Meier survival curves, the Low iron group had a much higher rate of composite events (log-rank P < 0.0001) (Fig. 1A). Similarly, in each of all-cause mortality and readmission for HF, the event rate was higher in the Low iron group (log-rank P < 0.0001 and P = 0.0003, respectively) (Fig. 1B,C). In addition, the composite event rates were higher in the Low iron group than the High iron group at any of 90-days, 6-months and 1-year. (Supplemental Fig. S1).

Table 2 shows unadjusted and adjusted HRs for composite outcomes in the two groups. Compared to the High iron group, the unadjusted HR for composite outcome was significantly higher in the Low iron group (HR 1.956; 95% CI 1.570–2.444; P < 0.0001). Even after adjustment for covariates (age, sex, LVEF, Hb, eGFR, BNP, TSAT, and ferritin level) in the multivariable Cox proportional hazards models, this finding remained significant (HR 1.500; 95% CI 1.128–1.976; P = 0.0044). Moreover, in a further analysis of factors related to anemia and iron balance, including hemoglobin, MCV, ferritin, transferrin level, and TSAT, the low iron group was significantly associated with composite outcomes (HR 1.487; 95% CI 1.074–2.059; P = 0.0169) (Table 2 Model 5). In this study, a total of 74 patients had a history of blood transfusion within 3 months before enrollment and during hospitalization. The analysis was performed excluding these patients, but the results were similar. (Supplemental Table S1) Moreover, the Low iron group was associated with composite outcomes with or without ID based on ferritin level defined as ferritin level < 100 ug/L or 100–299 ug/L if TSAT is < 20%. (Supplemental Table S2).

**Table 2:** Hazard ratios and 95% CI for composite events.

	All-cause death or readmission for HF	
	HR (95% CI)	P value
**Model 1**		
Low iron (serum Fe < 62 µg/dL)	1.956 (1.570–2.444)	< 0.0001
**Model 2**		
Low iron (serum Fe < 62 µg/dL)	1.739(1.392–2.179)	< 0.0001
Age, year	1.038 (1.027–1.049)	< 0.0001
Male	1.408 (1.128–1.757)	0.0023
**Model 3**		
Low iron (serum Fe < 62 µg/dL)	1.454 (1.149–1.847)	0.0020
Age, year	1.033 (1.022–1.044)	< 0.0001
Male	1.378 (1.100–1.726)	0.0053
Hemoglobin, g/dL	0.856 (0.800–0.916)	< 0.0001
eGFR, mL/min/1.73 m^2^	1.005 (0.999–1.010)	0.1004
Plasma BNP, 100 pg/mL	1.028 (1.006–1.048)	0.0082
LVEF, %	0.997 (0.989–1.004)	0.3747
**Model 4**		
Low iron (serum Fe < 62 µg/dL)	1.500 (1.128–1.976)	0.0044
Age, year	1.034 (1.023–1.047)	< 0.0001
Male	1.399 (1.108–1.766)	0.0047
Hemoglobin, g/dL	0.866 (0.807–0.928)	< 0.0001
eGFR, mL/min/1.73 m^2^	1.005 (0.999–1.011)	0.0930
Plasma BNP, 100 pg/Ll	1.026 (1.004–1.047)	0.0158
LVEF, %	0.996 (0.988–1.004)	0.3244
Ferritin, µg/L	1.000 (1.000–1.001)	0.5761
TSAT, %	1.001 (0.990–1.009)	0.8392
**Model 5**		
Low iron (serum Fe < 62 µg/dL)	1.487 (1.074–2.059)	0.0169
Hemoglobin, g/dL	0.856 (0.804–0.913)	< 0.0001
MCV, fl	1.038 (1.020–1.057)	< 0.0001
Ferritin, µg/L	1.000 (0.999–1.000)	0.4769
TSAT, %	0.998 (0.986–1.011)	0.7758
Transferrin, mg/dL	1.001 (0.998–1.004)	0.5442

*HF* heart failure; *eGFR* estimated glomerular filtration rate; *BNP* B-type natriuretic peptide; LVEF, left ventricular ejection fraction; *TSAT* transferrin saturation; *MCV* mean corpuscular volume; *HR* hazard ratio; *CI* confidence interval.

Based on ROC curve analysis, the serum iron cutoff value was 64 µg/dL in this study, with sensitivity of 66.1% and specificity of 58.2%. The area under the ROC curve was 0.6368. (Supplemental Fig. S2) The serum iron ≤ 64 ug/dl was an independent prognostic factor in multivariate analysis using Hemoglobin, BNP, and BUN that are well-known prognostic markers of mortality and readmission in HF. (Supplemental Table S3).

### HFrEF and HFpEF

**Figure 2:**
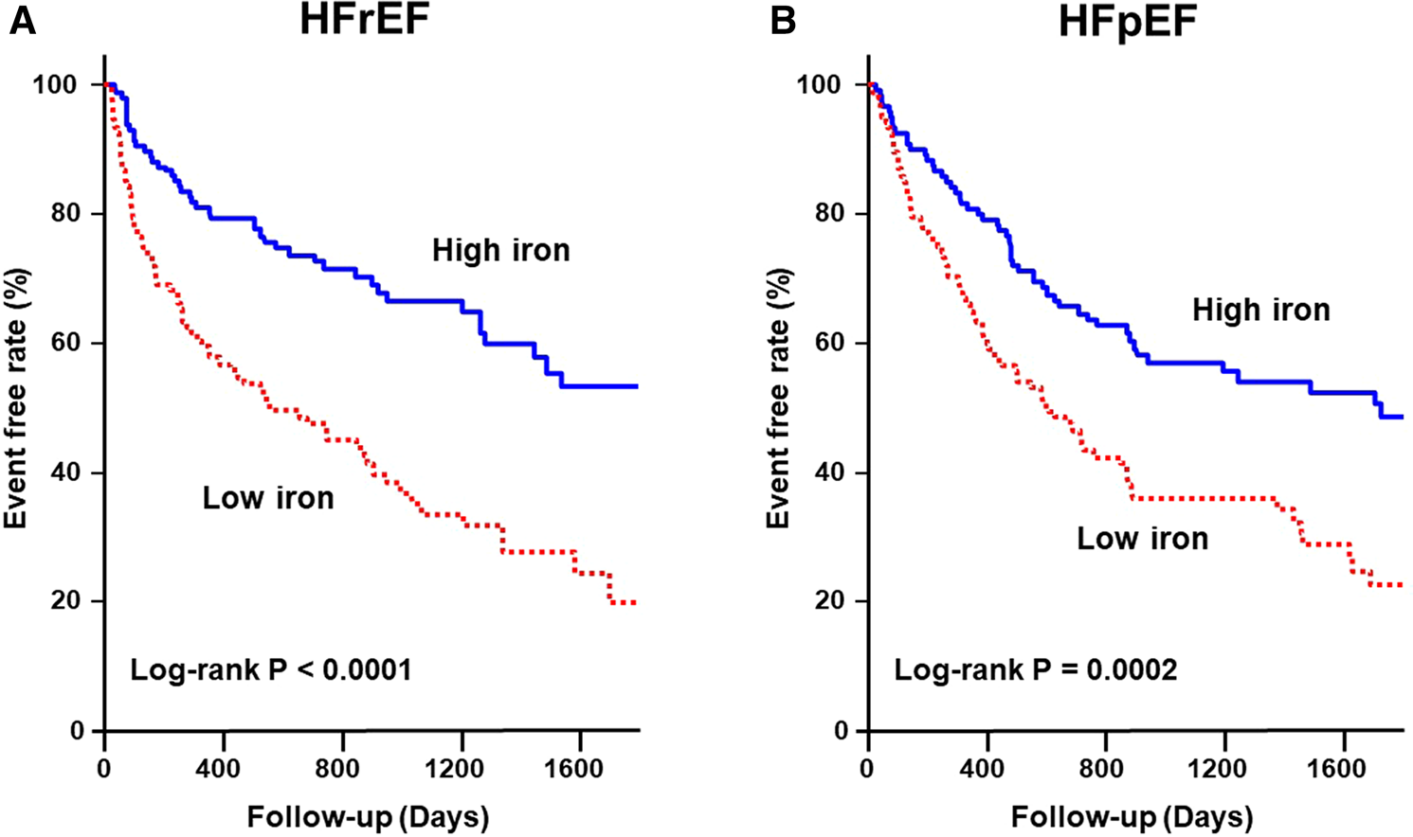
Kaplan–Meier event-free survival curves for all-cause mortality or readmission for HF in patients with HFrEF (**A**) and HFpEF (**B**) in the Low iron group (red line) compared to the High iron group (blue line). HFpEF, heart failure with preserved ejection fraction; HFrEF, heart failure with reduced ejection fraction.

Figure 2 shows the Kaplan–Meier survival curves for composite events in HFrEF and HFpEF. The Low iron group had a much higher rate of composite events in both patients with HFrEF and HFpEF (log-rank P < 0.0001 and P = 0.0002, respectively).

Table 3 shows unadjusted and adjusted HRs for composite outcomes in HFrEF and HFpEF. In both HFrEF and HFpEF, the unadjusted HR for composite outcome was significantly higher in the Low iron group (HR 2.404; 95% CI 1.661–3.515; P < 0.0001 and HR 1.886; 95% CI 1.349–2.657; P = 0.0002, respectively). In multivariable Cox proportional hazards models, the Low iron group was independently related to composite outcomes in both HFrEF and HFpEF (HR 1.564; 95% CI 1.018–2.402; P = 0.0411 and HR 1.769; 95% CI 1.094–2.860; P = 0.0201, respectively). Moreover, in an analysis of factors related to anemia and iron balance, the Low iron group tended to be associated with composite outcomes in both HFrEF and HFpEF.

**Table 3:** Hazard ratios and 95% CI for composite events in each of HFrEF and HFpEF.

	All-cause death or readmission for HF			
	HFrEF		HFpEF	
	HR (95% CI)	P value	HR (95% CI)	P value
**Model 1**				
Low iron (serum Fe < 62 µg/dL)	2.404 (1.661–3.515)	< 0.0001	1.886 (1.349–2.657)	0.0002
**Model 2**				
Low iron (serum Fe < 62 µg/dL)	2.082 (1.425–3.072)	0.0002	1.741 (1.243–2.456)	0.0014
Age, year	1.031 (1.012–1.051)	0.0014	1.037 (1.020–1.055)	< 0.0001
Male	1.637 (1.072–2.499)	0.0226	1.349 (0.967–1.881)	0.0776
**Model 3**				
Low iron (serum Fe < 62 µg/dL)	1.504 (1.006–2.269)	0.0467	1.615 (1.121–2.347)	0.0099
Age, year	1.019 (0.999–1.040)	0.0597	1.034 (1.017–1.051)	< 0.0001
Male	1.687 (1.090–2.611)	0.0148	1.358 (0.972–1.896)	0.0733
Hemoglobin, g/dL	0.798 (0.713–0.891)	< 0.0001	0.888 (0.791–0.994)	0.0391
eGFR, mL/min/1.73 m^2^	0.999 (0.987–1.010)	0.8059	1.007 (1.000–1.015)	0.0617
Plasma BNP, 100 pg/mL	1.022 (0.987–1.055)	0.2122	1.022 (0.974–1.061)	0.3450
LVEF, %	0.976 (0.949–1.005)	0.0972	1.007 (0.983–1.032)	0.5614
**Model 4**				
Low iron (serum Fe < 62 µg/dL)	1.564 (1.018–2.402)	0.0411	1.769 (1.094–2.860)	0.0201
Age, year	1.019 (0.999–1.041)	0.0639	1.036 (1.018–1.055)	< 0.0001
Male	1.725 (1.100–2.706)	0.0139	1.343 (0.952–1.894)	0.0930
Hemoglobin, g/dL	0.796 (0.709–0.891)	< 0.0001	0.898 (0.798–1.011)	0.0747
eGFR, mL/min/1.73 m^2^	0.999 (0.988–1.010)	0.8755	1.008 (0.999–1.016)	0.0662
Plasma BNP, 100 pg/mL	1.019 (0.982–1.054)	0.3052	1.023 (0.980–1.067)	0.3029
LVEF, %	0.975 (0.948–1.004)	0.0906	1.006 (0.981–1.032)	0.6293
Ferritin, µg/L	1.000 (0.999–1.001)	0.8929	1.000 (0.999–1.001)	0.8627
TSAT, %	1.003 (0.991–1.011)	0.5676	1.007 (0.989–1.025)	0.4461
**Model 5**				
Low iron (serum Fe < 62 µg/dL)	1.638 (1.037–2.563)	0.0311	1.726 (0.998–2.985)	0.0508
Hemoglobin, g/dL	0.808 (0.725–0.900)	0.0001	0.882 (0.777–1.001)	0.0515
MCV, fl	1.060 (1.025–1.097)	0.0008	1.020 (0.992–1.050)	0.1674
Ferritin, µg/L	0.999 (0.998–1.001)	0.4057	1.000 (0.999–1.001)	0.8305
TSAT, %	1.002 (0.985–1.011)	0.7454	1.002 (0.982–1.022)	0.8496
Transferrin, mg/dL	0.997 (0.993–1.002)	0.2227	1.005 (1.000–1.009)	0.0327

*HF* heart failure; *HFrEF* heart failure with reduced ejection fraction; *HFpEF* heart failure with preserved ejection fraction; *eGFR* estimated glomerular filtration rate; *BNP* B-type natriuretic peptide; *LVEF* left ventricular ejection fraction; *TSAT* transferrin saturation; *MCV* mean corpuscular volume, *HR* hazard ratio; *CI* confidence interval.

### Factors related to low iron levels

In the analysis according to odds ratio, age, diabetes mellitus, CRP, eGFR, and BNP were related to low iron levels. Multivariate analysis using these factors revealed that all factors were independently associated with low iron levels (Supplemental Table S4).

## Discussion

The present study investigated the significance of serum iron levels in patients with ADHF and demonstrated that serum iron level is independently associated with poor prognosis in patients with ADHF. To the best of our knowledge, this is the first report demonstrating an association of serum iron level per se with the prognosis of patients with ADHF. Compared to previous studies that investigated the relationship between ID based on ferritin level and HF, this study has several new advantages. First, the target patients had ADHF, not chronic HF. Second, we studied serum iron levels instead of ferritin levels. Finally, a low iron level was associated with adverse outcomes in both HFrEF and HFpEF.

Clinical and prognostic significance of ID in patients with HF has previously been recognized^1, 8, 15^. ID is associated with reduced exercise capacity, 6-min walk test values, and peak oxygen consumption^13, 16^, as well as poor prognosis and quality of life in patients with chronic HF^13, 14, 17^. Generally, ID is defined as ferritin level < 100 ug/L or 100–299 ug/L if TSAT is < 20%^15, 18, 19^. However, in patients with ADHF, it may be difficult to apply the ID criteria based on this definition due to the impaired iron action^20^. One of the reasons for impaired iron action is the effect of hepcidin. Hepcidin acts as a major homeostatic regulator of iron metabolism by blocking ferroportin, which is the only known mammalian iron exporting protein^21, 22^. ADHF is associated with a systemic inflammatory response whose sustained level over time is not linked to the decrease in mechanical myocardial stress, suggesting the unique role of inflammation in acute HF pathophysiology^10, 11^. Consequently, in patients with ADHF, the increasing hepcidin activity due to inflammation reduces duodenal iron absorption and simultaneously reduces iron release from stores in the reticulo-endothelial cells and hepatocytes, thereby causing functional ID. Our finding that CRP was associated with low iron levels supports this theory. Therefore, despite the widely accepted and reported above definitions in chronic HF, using ferritin as an indicator of functional ID may be challenging in patients with ADHF. Given that serum iron acts on the tissue, it can be argued that this is a more physiologically relevant variable; herein, we found a correlation between poor prognosis and low iron level. Notably, low iron level was an independent predictor of adverse outcomes even in multivariate analysis including hemoglobin and MCV. This finding is consistent with that from previous reports, which showed that ID was associated with poor prognosis in chronic HF, irrespective of whether anemia is present^7, 13, 14, 17^. These findings may imply that the significance of iron is shifting from the cause of anemia toward the more direct effects of iron itself on non-hematopoietic tissues such as cardiac muscle and skeletal muscles^23^. The oxygen-transport properties of hemoglobin depend on the binding of oxygen to ferrous iron within heme. Within the mitochondrion, iron is an essential component of several subunits of the electron transport chain. Recently, it was reported that augmented skeletal muscle energetics might be an important mechanism via which iron repletion confers benefits in HF despite minimal hemoglobin changes^24^. Therefore, serum iron may have important roles unrelated to anemia.

There are multiple mechanisms in addition to the action on hepcidin whereby serum iron levels may decrease in patients with HF, including excitation of the sympathetic nervous system, reduction of iron absorption by intestinal wall edema, and adverse effects from prescribed drugs such as histamine-2 receptor antagonists and proton-pump inhibitors^25^. Anemia due to ID reduces the ability of hemoglobin to transport oxygen and causes hypoxemia. Therefore, from the viewpoint of cardio-renal-anaemia syndrome, iron administration may be useful, especially in HF with anemia. In this study, the serum iron cutoff value was 64 µg/dL based on ROC curve analysis. Therefore, it may be better to consider intravenous iron therapy for the serum iron ≤ 64 µg/dL. Of course, large-scale studies and intervention studies are needed to set an appropriate reference value. In Japan, the length of hospital stay for HF is often longer than in other countries. Therefore, in patients with HF who have a short hospital stay, it may be necessary to pay attention to low iron not only at the time of discharge but also after discharge.

Moreover, in this study, low iron level was a prognostic factor in patients with HFrEF and HFpEF. Regarding HFrEF, the effectiveness of intravenous iron administration for chronic HF has been previously reported^12, 26, 27^. Our results suggest that intravenous iron administration may be useful even in the acute phase of HFrEF. However, caution is required to prevent iron overload. Some studies have demonstrated that iron overload can increase the risk of adverse cardiovascular events, such as HF and cardiomyopathy^28^. Therefore, frequent administration of iron agents should be avoided; in each case, it is necessary to carefully confirm the indications for iron administration. On the other hand, our findings suggest that iron administration may be a new therapeutic target in HFpEF. As is generally known, no useful treatment has been demonstrated for HFpEF. Therefore, it is a landmark finding if the efficacy of iron administration is demonstrated. However, this needs to be examined further in large-scale studies.

### Limitations

There are several limitations to this study. First, the sample size was moderate, and the study was conducted at a single center. Second, we did not collect hepcidin, ferroportin, or biomarkers for inflammation, such as calcitonin or red cell distribution width except for CRP. Therefore, we could not directly examine hepcidin activity in this study population. Third, we had no data on other factors that influence hepcidin. Fourth, among the eligible study population, many patients were excluded in this study due to in-hospital deaths or absence of serum iron measurement at discharge. Although there was no difference between the baseline of the entire study patients and the baseline of this study patients, this may be a potential bias in the results.

## Conclusion

We demonstrated that low iron level was an independent poor prognostic factor in ADHF, irrespective of hemoglobin or ferritin level. In acute HF, it is important to focus not only on ferritin but also on iron itself. Although further large-scale studies are warranted to verify the present results, our findings highlight a novel potential for iron replacement as a therapeutic target to further reduce adverse events in patients with HFpEF.

## Methods

### Patient selection

The NARA-HF study is a prospective and dynamic cohort study^29, 30^. The NARA-HF 4 study recruited 1012 consecutive patients emergently admitted to the cardiology wards or the coronary care unit at our hospital for the first time due to ADHF between January 2011 and December 2018. The diagnosis of HF was based on the Framingham criteria^31^. Patients with acute myocardial infarction (AMI), acute myocarditis, and acute HF with acute pulmonary embolism were excluded. Furthermore, re-registration at the time of readmission will not be performed.

Of the 1012 patients, 615 underwent serum iron measurement at discharge. Patients were divided into low (the Low iron group, n = 307) and high (the High iron group, n = 308) serum iron groups based on the median serum iron level (62 µg/dL). Baseline data for each patient included age, sex, body mass index (BMI), cause of HF, medical history, vital signs, laboratory and echocardiographic data, and medications on admission and at discharge.

The study was approved by the Ethics Committee of Nara Medical University, and written informed consent was obtained from all patients in accordance with the Declaration of Helsinki’s Ethical Principles for Medical Research Involving Human Subjects.

### Outcomes

The endpoint was set as the composite outcome, which included all-cause mortality and readmission for HF. Medical records were reviewed to determine vital status and the cause of death. When this information was unavailable in the medical record, we telephoned patients or their families. Information regarding cardiovascular events such as non-fatal AMI, stroke, and re-hospitalization due to recurrence of ADHF was also obtained. Furthermore, we reanalyzed these examinations in each case of HFrEF and HFpEF.

### Statistical analysis

Continuous variables were expressed as mean ± standard deviation and compared using Student’s t-test. Categorical variables were summarized with frequency percentages and analyzed using a chi-square test. Cumulative event-free rates during follow-up were derived using the Kaplan–Meier method. Univariate and multivariable analyses of events were performed using Cox proportional hazards models. We implemented five models for the adjustment of covariates: model 1, unadjusted; model 2, adjusted for age and sex; model 3, adjusted for all factors in model 2 plus hemoglobin, estimated glomerular filtration rate (eGFR), brain natriuretic peptide (BNP), and LVEF; model 4, adjusted for all factors in model 3 plus ferritin level and transferrin saturation (TSAT); and model 5, adjusted for factors related to anemia and iron balance including hemoglobin, mean corpuscular volume (MCV), ferritin, transferrin level, and TSAT.

Results were reported as hazard ratios (HR), 95% confidence intervals (CI), and P-values. The HR for outcomes in the Low iron group was compared with that for outcomes in the High iron group, which served as the reference group. Variables with P-values < 0.05 were retained in the model. JMP version 14 for Windows (SAS Institute, Cary, NC, USA) was used for all statistical analyses.

## Supplementary Information

Supplementary Figure 1.

Supplementary Figure 2.

Supplementary Table 1.

Supplementary Table 2.

Supplementary Table 3.

Supplementary Table 4.

Supplementary Figures Legend.
