# Supplementary material for: Serum iron: a new predictor of adverse outcomes independently from serum hemoglobin levels in patients with acute decompensated heart failure
Source: Sci Rep. 2021 Jan 27;11:2395. doi: 10.1038/s41598-021-82063-0 (PMC7840917; doi:10.1038/s41598-021-82063-0)
Supplement: Supplementary file 1 — Supplementary Figure 1. [file 41598_2021_82063_MOESM1_ESM.pptx]

## Slide 1
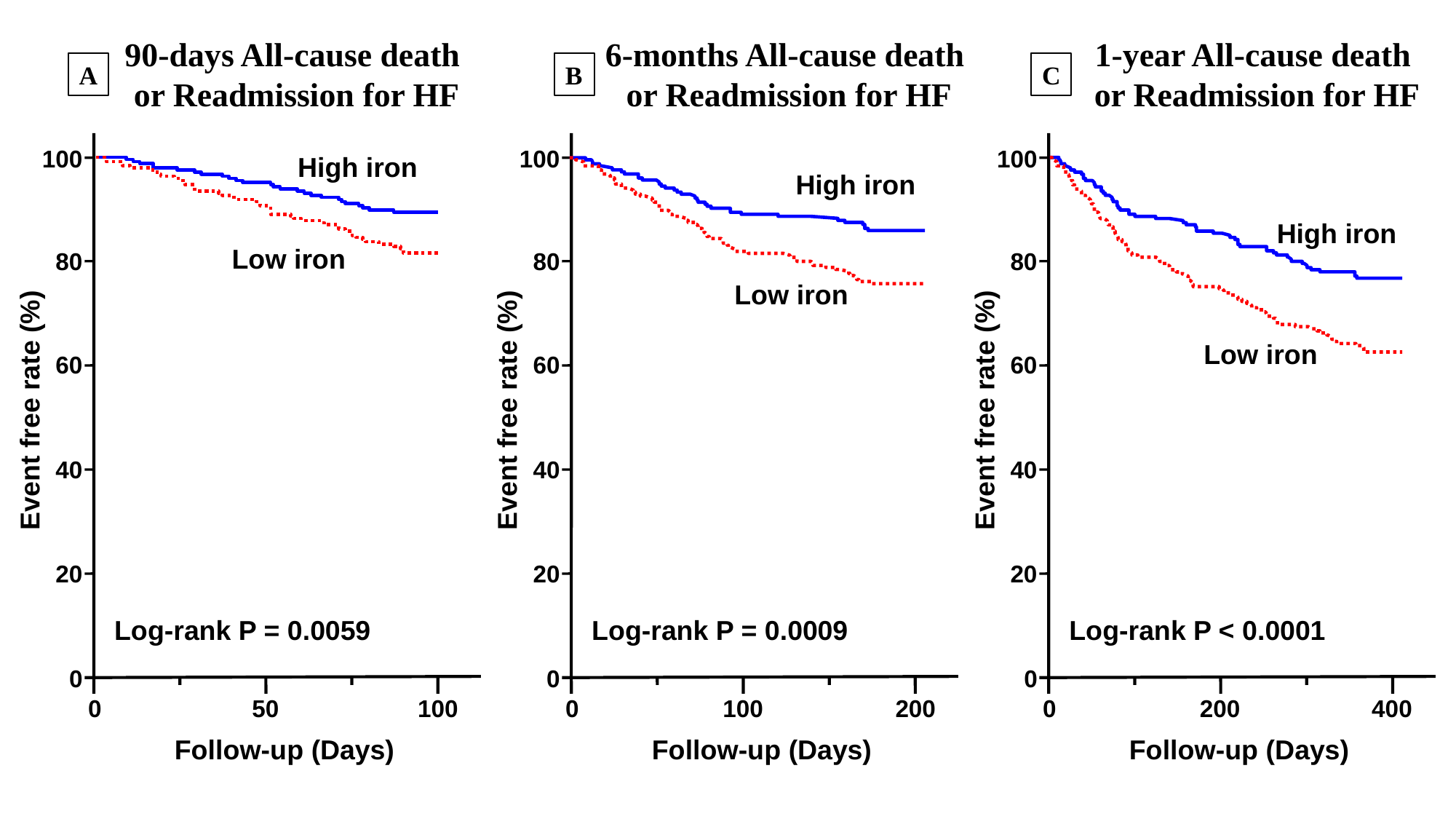

90-days All-cause death
 or Readmission for HF
6-months All-cause death
 or Readmission for HF
1-year All-cause death
 or Readmission for HF
A
B
C
100
High iron
Event free rate (%)
Low iron
80
60
40
20
Log-rank P = 0.0059
0
0
50
100
Follow-up (Days)
100
High iron
Event free rate (%)
80
Low iron
60
40
20
Log-rank P = 0.0009
0
0
100
200
Follow-up (Days)
100
Event free rate (%)
High iron
80
Low iron
60
40
20
Log-rank P < 0.0001
0
0
200
400
Follow-up (Days)
