# Supplementary figures and images for: Serum iron: a new predictor of adverse outcomes independently from serum hemoglobin levels in patients with acute decompensated heart failure
Source: Sci Rep. 2021 Jan 27;11:2395. doi: 10.1038/s41598-021-82063-0 (PMC7840917; doi:10.1038/s41598-021-82063-0)

## Slide 1
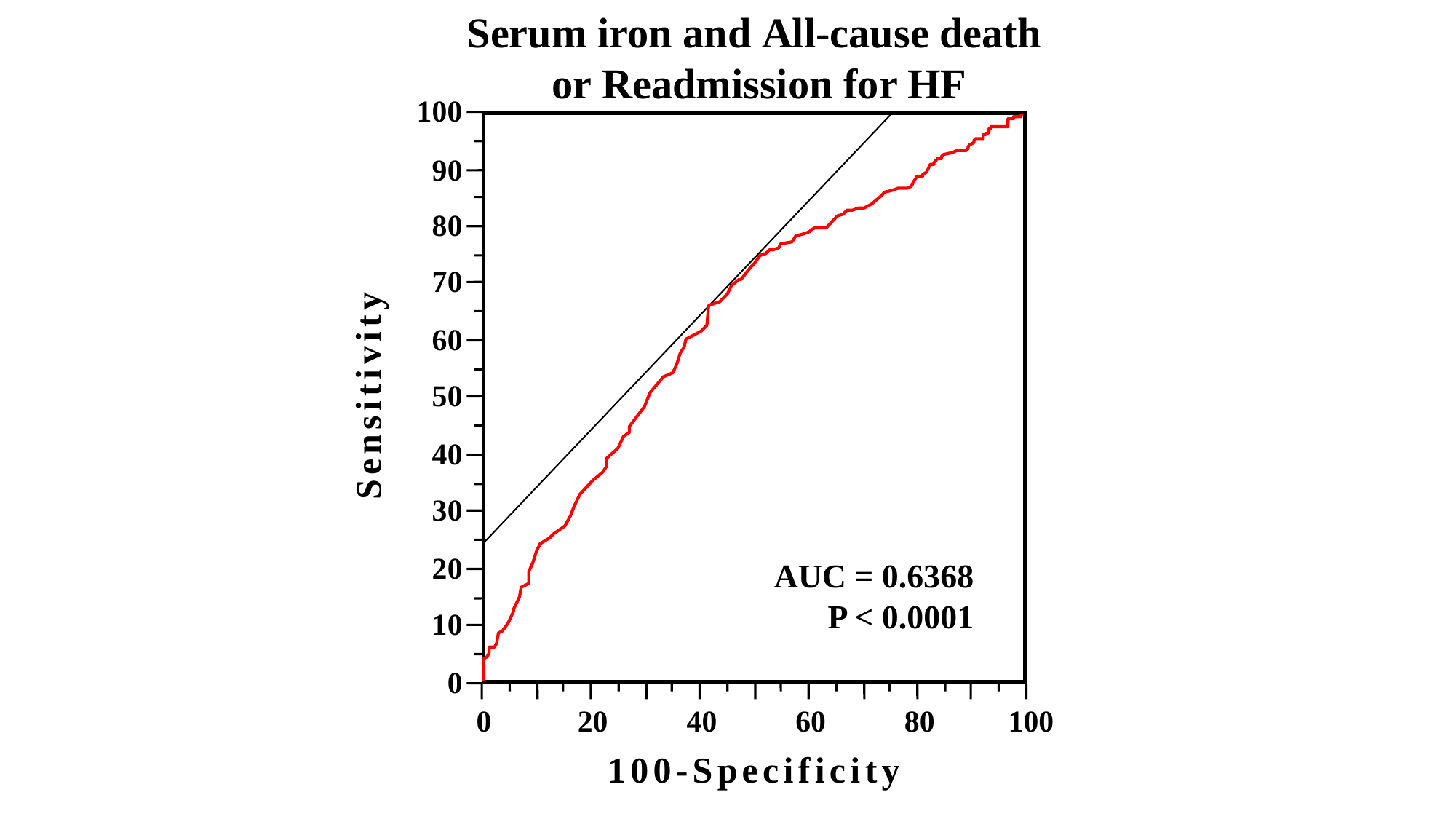

Serum iron and All-cause death
 or Readmission for HF
100
90
80
70
60
Sensitivity
50
40
30
20
AUC = 0.6368
P < 0.0001
10
0
0
20
40
60
80
100
100-Specificity

Supplement: Supplementary file 2 — Supplementary Figure 2. [file 41598_2021_82063_MOESM2_ESM.pptx]
