# Supplementary material for: Serum iron: a new predictor of adverse outcomes independently from serum hemoglobin levels in patients with acute decompensated heart failure
Source: Sci Rep. 2021 Jan 27;11:2395. doi: 10.1038/s41598-021-82063-0 (PMC7840917; doi:10.1038/s41598-021-82063-0)
Supplement: Supplementary file 3 — Supplementary Table 1. [file 41598_2021_82063_MOESM3_ESM.docx]

Supplemental Table 1. Hazard ratios and 95% CI for composite events except for patients with transfusion history

|  | **All-cause death or readmission for HF** | |
| --- | --- | --- |
| **Model 1** | **HR (95% CI)** | **P value** |
| **Low iron (serum Fe < 62 µg/dL)** | 2.039 (1.605–2.589) | <0.0001 |

|  | **All-cause death or readmission for HF** | |
| --- | --- | --- |
| **Model 2** | **HR (95% CI)** | **P value** |
| **Low iron (serum Fe < 62 µg/dL)** | 1.814 (1.422–2.313) | <0.0001 |
| **Age, year** | 1.035 (1.023–1.047) | <0.0001 |
| **Male** | 1.434 (1.126–1.826) | 0.0034 |

|  | **All-cause death or readmission for HF** | |
| --- | --- | --- |
| **Model 3** | **HR (95% CI)** | **P value** |
| **Low iron (serum Fe < 62 µg/dL)** | 1.519 (1.172–1.968 | 0.0016 |
| **Age, year** | 1.031 (1.019–1.043) | <0.0001 |
| **Male** | 1.385 (1.079–1.777) | 0.0106 |
| **Hemoglobin, g/dL** | 0.880 (0.819–0.945) | 0.0005 |
| **eGFR, ml/min/1.73m^2^** | 1.003 (0.997–1.010) | 0.3222 |
| **Plasma BNP, 100 pg/mL** | 1.025 (1.003–1.047) | 0.0259 |
| **LVEF, %** | 0.996 (0.988–1.005) | 0.3961 |

|  | **All-cause death or readmission for HF** | |
| --- | --- | --- |
| **Model 4** | **HR (95% CI)** | **P value** |
| **Low iron (serum Fe < 62 µg/dL)** | 1.514 (1.113–2.059) | 0.0083 |
| **Age, year** | 1.033 (1.020–1.045) | <0.0001 |
| **Male** | 1.433 (1.107–1.855) | 0.0064 |
| **Hemoglobin, g/dL** | 0.891 (0.828–0.959) | 0.0021 |
| **eGFR, ml/min/1.73m^2^** | 1.003 (0.997–1.010) | 0.3295 |
| **Plasma BNP, 100 pg/mL** | 1.024 (1.001–1.047) | 0.0413 |
| **LVEF, %** | 0.996 (0.988–1.004) | 0.3579 |
| **Ferritin, µg/L** | 1.000 (1.000–1.001) | 0.5607 |
| **TSAT, %** | 0.999 (0.989–1.010) | 0.8600 |

|  | **All-cause death or readmission for HF** | |
| --- | --- | --- |
| **Model 5** | **HR (95% CI)** | **P value** |
| **Low iron (serum Fe < 62 µg/dL)** | 1.539 (1.076–2.159) | 0.0151 |
| **Hemoglobin, g/dL** | 0.875 (0.813–0.940) | 0.0003 |
| **MCV, fl** | 1.038 (1.017–1.060) | 0.0003 |
| **Ferritin, µg/L** | 1.000 (0.999–1.000) | 0.3912 |
| **TSAT, %** | 0.996 (0.980–1.007) | 0.5754 |
| **Transferrin, mg/dL** | 1.001 (0.998–1.003) | 0.6856 |

HF, heart failure; eGFR, estimated glomerular filtration rate; BNP, B-type natriuretic peptide;

LVEF, left ventricular ejection fraction; TSAT, transferrin saturation; MCV, mean corpuscular volume;

HR, hazard ratio; CI, confidence interval.
