# Supplementary material for: Serum iron: a new predictor of adverse outcomes independently from serum hemoglobin levels in patients with acute decompensated heart failure
Source: Sci Rep. 2021 Jan 27;11:2395. doi: 10.1038/s41598-021-82063-0 (PMC7840917; doi:10.1038/s41598-021-82063-0)
Supplement: Supplementary file 4 — Supplementary Table 2. [file 41598_2021_82063_MOESM4_ESM.docx]

Supplemental Table 2. Hazard ratios and 95% CI for composite events in each of patients with or without ID based on ferritin level

|  | **All-cause death or readmission for HF** | | | |
| --- | --- | --- | --- | --- |
|  | **Without ID based on Ferritin level** | | **With ID based on Ferritin level** | |
|  | **HR (95% CI)** | **P value** | **HR (95% CI)** | **P value** |
| **Low iron (serum Fe < 62 µg/dL)** | 1.665 (1.054–2.640) | 0.0291 | 1.778 (1.057–2.988) | 0.0299 |
| **Age, year** | 1.030 (1.009–1.052) | 0.0064 | 1.037 (1.021–1.053) | <0.0001 |
| **Male** | 1.215 (0.782–1.886) | 0.3866 | 1.447 (1.075–1.948) | 0.0149 |
| **Hemoglobin, g/dL** | 0.872 (0.775–0.979) | 0.0212 | 0.889 (0.805–0.982) | 0.0204 |
| **eGFR, ml/min/1.73m^2^** | 1.002 (0.991–1.013) | 0.6964 | 1.006 (0.999–1.014) | 0.1010 |
| **Plasma BNP, 100 pg/mL** | 1.019 (0.980–1.055) | 0.3093 | 1.023 (0.995–1.051) | 0.1094 |
| **LVEF, %** | 0.993 (0.979–1.006) | 0.2855 | 0.997 (0.987–1.007) | 0.5742 |
| **Ferritin, µg/L** | 1.000 (1.000–1.001) | 0.2674 | 1.001 (0.998–1.014) | 0.6357 |
| **TSAT, %** | 1.002 (0.990–1.010) | 0.6187 | 0.992 (0.971–1.014) | 0.4809 |

HF, heart failure; ID, iron deficiency; eGFR, estimated glomerular filtration rate; BNP, B-type natriuretic peptide; LVEF, left ventricular ejection fraction; TSAT, transferrin saturation.

HR, hazard ratio; CI, confidence interval.

ID based on ferritin level is defined as ferritin level <100 ug/L or 100-299 ug/L if TSAT is <20%.
