# Supplementary material for: Serum iron: a new predictor of adverse outcomes independently from serum hemoglobin levels in patients with acute decompensated heart failure
Source: Sci Rep. 2021 Jan 27;11:2395. doi: 10.1038/s41598-021-82063-0 (PMC7840917; doi:10.1038/s41598-021-82063-0)
Supplement: Supplementary file 5 — Supplementary Table 3. [file 41598_2021_82063_MOESM5_ESM.docx]

Supplemental Table 3. Hazard ratios and 95% CI for composite events

|  | **All-cause death or readmission for HF** | |
| --- | --- | --- |
|  | **HR (95% CI)** | **P value** |
| **Low iron (serum Fe ≤ 64 mg/dl)** | 1.551 (1.219–1.986) | 0.0003 |
| **Hemoglobin, g/dl** | 0.875 (0.821–0.933) | <0.0001 |
| **Plasma BNP, 100 pg/ml** | 1.028 (1.008–1.046) | 0.0062 |
| **BUN, mg/dl** | 1.009 (1.002–1.014) | 0.0066 |

HF, heart failure; BNP, B-type natriuretic peptide; BUN, blood urea nitrogen.

HR, hazard ratio; CI, confidence interval.
