# Supplementary material for: Serum iron: a new predictor of adverse outcomes independently from serum hemoglobin levels in patients with acute decompensated heart failure
Source: Sci Rep. 2021 Jan 27;11:2395. doi: 10.1038/s41598-021-82063-0 (PMC7840917; doi:10.1038/s41598-021-82063-0)
Supplement: Supplementary file 6 — Supplementary Table 4. [file 41598_2021_82063_MOESM6_ESM.docx]

Supplemental Table 4. Factors related to Low iron

|  | **Univariate** | | **Multivariate** | |
| --- | --- | --- | --- | --- |
|  | **OR (95% CI)** | **P value** | **OR (95% CI)** | **P value** |
| **Age, years** | 1.030 (1.016-1.045) | <0.0001 | 1.019 (1.004-1.035) | 0.0144 |
| **Diabetes mellitus** | 1.634 (1.182-2.259) | 0.0028 | 1.519 (1.068-2.160) | 0.0200 |
| **CRP, mg/dl** | 1.667 (1.366-2.035) | <0.0001 | 1.596 (1.308-1.947) | <0.0001 |
| **eGFR, ml/min/1.73m^2^** | 0.977 (0.970-0.985) | <0.0001 | 0.981 (0.973-0.989) | <0.0001 |
| **BNP, 100pg/ml** | 1.084 (1.039-1.132) | <0.0001 | 1.045 (1.003-1.095) | 0.0370 |

CRP, C-reactive protein; eGFR, estimated glomerular filtration rate; BNP, B-type natriuretic peptide

OR, Odds ratio; CI, confidence interval.
