# Supplementary material for: Serum iron: a new predictor of adverse outcomes independently from serum hemoglobin levels in patients with acute decompensated heart failure
Source: Sci Rep. 2021 Jan 27;11:2395. doi: 10.1038/s41598-021-82063-0 (PMC7840917; doi:10.1038/s41598-021-82063-0)
Supplement: Supplementary file 7 — Supplementary Figures Legend. [file 41598_2021_82063_MOESM7_ESM.docx]

**Supplemental Figure 1.** Kaplan-Meier event-free survival curves for all-cause mortality or readmission for HF in the Low iron group (red line) compared with the High iron group (blue line) (A) in 90-day, (B) in 6-months, and (C) in 1-year.

HF, heart failure

**Supplemental Figure 2.** ROC curve analysis for Serum iron and All-cause death or Readmission for HF. At the optimal cut-off of the serum iron 64 µg/dL, sensitivity was 66.1% and specificity was 58.2%. The area under the curve was 0.6368.
